# Free-electron coupling to surface polaritons mediated by small scatterers

**DOI:** 10.1515/nanoph-2024-0326

**Published:** 2024-10-04

**Authors:** Leila Prelat, Eduardo J. C. Dias, F. Javier García de Abajo

**Affiliations:** ICFO-Institut de Ciencies Fotoniques, The Barcelona Institute of Science and Technology, 08860 Castelldefels, Barcelona, Spain; ICREA-Institució Catalana de Recerca i Estudis Avançats, Passeig Lluís Companys 23, 08010, Castelldefels, Barcelona, Spain

**Keywords:** nanophotonics, free electrons, polaritonic Smith–Purcell emission, coupling to confined polaritons

## Abstract

The ability of surface polaritons (SPs) to enhance and manipulate light fields down to deep-subwavelength length scales enables applications in optical sensing and nonlinear optics at the nanoscale. However, the wavelength mismatch between light and SPs prevents direct optical excitation of surface-bound modes, thereby limiting the widespread development of SP-based photonics. Free electrons are a natural choice to directly excite strongly confined SPs because they can supply field components of high momentum at designated positions with subnanometer precision. Here, we theoretically explore free-electron–SP coupling mediated by small scatterers and show that low-energy electrons can efficiently excite surface modes with a maximum probability reached at an optimum surface–scatterer distance. By aligning the electron beam with a periodic array of scatterers placed near a polariton-supporting interface, in-plane Smith–Purcell emission results in the excitation of surface modes along well-defined directions. Our results support using scattering elements to excite SPs with low-energy electrons.

## 1 Introduction

Surface polaritons (SPs) are collective charge oscillations occurring at the interfaces of a wide range of materials and offering a platform to control light–matter interactions at the nanoscale [1], [2]. Specifically, surface-bound optical modes allow us to concentrate electromagnetic energy down to deep-subwavelength length scales and produce large enhancements of the associated near electric fields [3]. Polaritons in two-dimensional (2D) materials are particularly promising because they are highly confined [4], [5] and can be widely controlled by external stimuli introduced through electrical gating [6], [7], [8], [9], chemical doping [10], [11], magnetic fields [12], or optical heating [13], [14], [15], [16]. These appealing properties enable technological applications in areas such as biosensing [17], [18], [19], [20], photodetection [21], [22], [23], light harvesting [24], [25], nonlinear optics [15], [26], [27], [28], and optoelectronics [29], [30], [31].

Unfortunately, strong field confinement in SPs comes at a price, as these modes cannot be directly excited by far-field radiation due to the kinematical mismatch that maintains them trapped to the surface. This problem limits practical applications of SPs, so much effort has been devoted to tackling it through different approaches, including the use of prisms [32] and nanotips [33], [34]. However, prisms are impractical when the SP in-plane wavelength *λ*_p_ is small compared with the light wavelength *λ*_0_ = 2*πc*/*ω* at the same frequency *ω* because of the lack of materials with sufficiently high refractive index $>\lambda_{0}/\lambda_{\text{p}}\gg 1$ . In addition, coupling elements such as gratings [35], while very efficient at exciting and controlling SPs, unavoidably modify the SP characteristics and offer poor spatial precision. Alternatively, small coupling nanostructures such as tips [36] are very efficient in focusing light [37], [38], but comparatively inefficient at launching SPs [39] unless strong conditions are met including a precise angular profile of the external light [40].

As an alternative, electron beams (e-beams) combine high spatial precision with an efficient coupling to SPs [41]. These probes were instrumental in pioneering studies of surface plasmons [42], [43] and are currently reaching a simultaneous spectral/spatial resolution in the few-meV/sub-nm range using techniques such as electron energy-loss spectroscopy [44], [45], [46], [47], [48], [49], [50], [51], [52], [53], [54] (EELS) and photon-induced near-field electron microscopy (PINEM) [55], [56], [57], [58], [59], [60], [61], [62], [63], [64], [65], [66], [67], [68], [69]. The detection of cathodoluminescence light emission resulting from the e-beam interaction with a specimen at a nanometer-controlled position also permits studying bright modes with a spectral resolution depending on the optical spectrometer and the signal-to-noise ratio [70], [71]. Recently, within the emerging field of ultrafast electron microscopy, a temporal resolution in the sub-fs regime [58], [72], [73] has become a possibility by teaming up pulsed lasers and free electrons, while also enabling electron spectromicroscopy to be performed with sub-nm/sub-meV resolution [74], [75]. It should be noted that e-beams effectively act as broadband nanoscale optical sources and, therefore, lack mode selectivity. In particular, an electron moving with velocity *v* parallel to a polariton-supporting planar surface can excite a wide spectrum of modes satisfying the condition *ω* < *k*_‖_ *v*, where *ω* and *k*_‖_ are the mode frequency and total in-plane wave vector, respectively. In this configuration, the emission is delocalized along the electron interaction path, and relativistic electrons are needed to excite SPs whose dispersion lies close to the light cone. These limitations could be potentially addressed by combining electron excitation with small structures placed in the vicinity of the surface and acting as coupling elements.

In this article, we theoretically explore the excitation of SPs in planar surfaces by parallel e-beams assisted by small scatterers. Specifically, we obtain a clean SP signal by operating under conditions in which electrons cannot directly excite surface modes (i.e., for *ω* > *k*_‖_ *v*), so that the scatterers act as intermediate elements where a dipole is induced containing the high-momentum components that are needed to excite SPs. With this approach, narrow-band SP emission can be realized if the scatterer features a spectrally localized mode. We demonstrate that the SP excitation efficiency can be maximized when the scatterer is placed at an optimum distance from the surface, as illustrated by studying the generation of surface-plasmon polaritons (SPPs) in a thin film (e.g., graphene and atomically thin metal layers). Perfectly resonant scatterers and hBN disks are considered. The latter constitute a practical realization of deep-subwavelength resonant scatterers operating in the mid-infrared spectral region. We further extend the phenomenon of Smith–Purcell emission [76] from photons to polaritons by placing a periodic particle array above a polariton-supporting surface and exciting it by a parallel e-beam, leading to the generation of collimated SPs at angles depending on the periodicity, the electron velocity, and the mode dispersion relation. Like in the individual particle, an optimum scatterer–surface distance is also obtained for polaritonic Smith–Purcell emission. These results pave the way for using free electrons combined with small scatterers to control the generation of polaritons in planar surfaces.

## 2 Electron excitation of surface polaritons mediated by a dipolar particle

**Figure 1:**
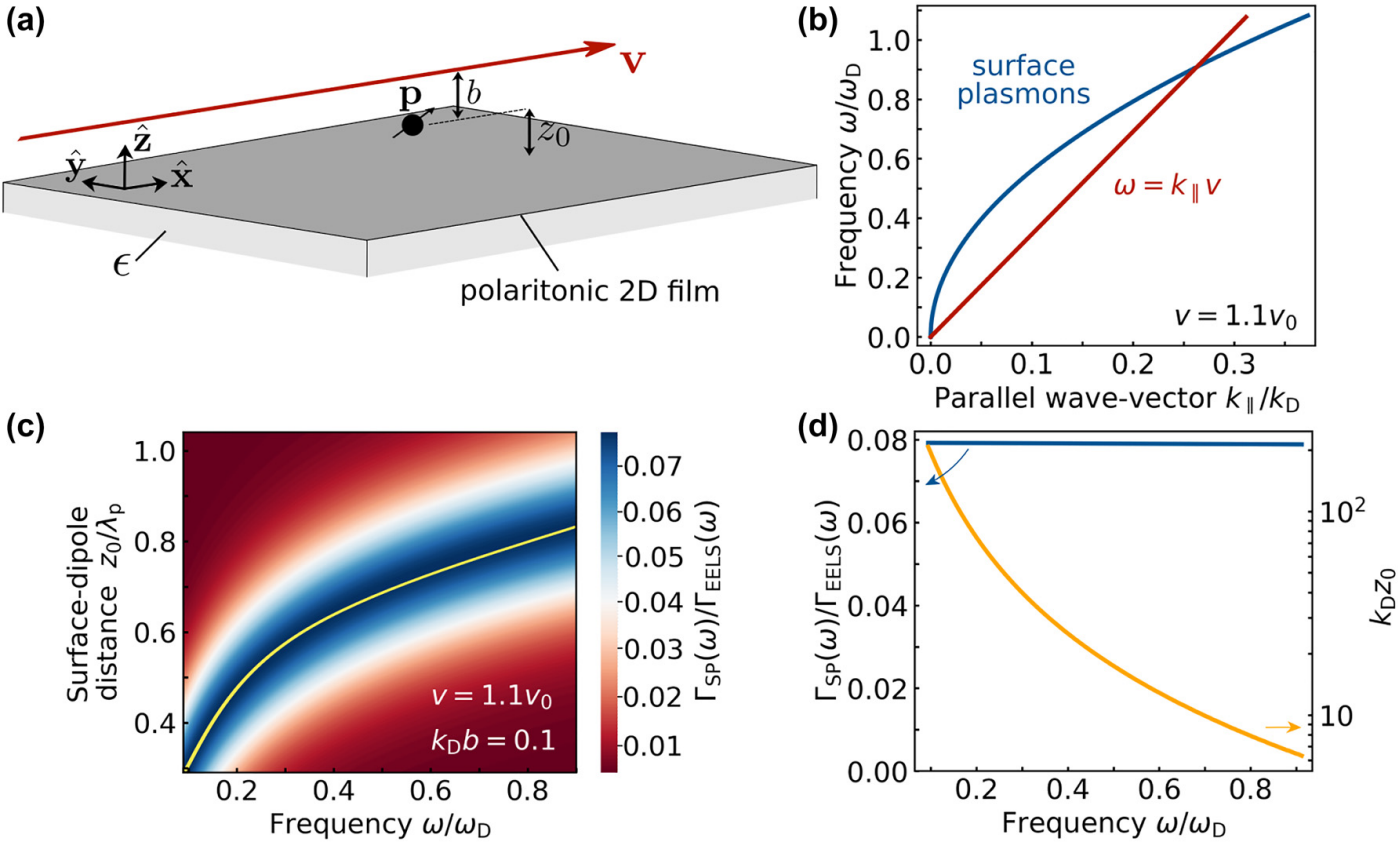
Free-electron excitation of surface polaritons (SPs) mediated by a dipolar scatterer. (a) Geometry under consideration, consisting of an electron moving with velocity **v** parallel to a polariton-supporting surface (a film placed at *z* = −*z*_0_ supported on a substrate of real permittivity *ϵ*) and passing above a small particle placed at **r** = 0 (at a distance *b* from the trajectory and *z*_0_ from the surface). The electron induces a dipole **p** on the particle, which couples to the SPs. The particle is in the plane defined by **v** and the surface normal. (b) Dispersion relation of surface-plasmon polaritons (SPPs) $\omega /\omega_{\text{D}}=\sqrt{k_{\|}/k_{\text{D}}}$ for a 2D film described by a Drude conductivity of weight *ω*_D_ [Eq. (10)] with *k*_D_ = (*ϵ* + 1)*ℏω*_D_/4*πe* ^2^ (e.g., *ω*_D_ = *E*_F_/*πℏ* for graphene) compared with the electron line *ω* = *k*_‖_ *v*. Both curves intersect at *ω*/*ω*_D_ = *v*_0_/*v*, where *v*_0_ = *ω*_D_/*k*_D_ ≈ 0.092 *c*/(*ϵ* + 1). We take *v* = 1.1 *v*_0_. (c) Spectrally resolved probability of SPP emission Γ_SP_(*ω*) under the conditions of (a, b) as a function of excitation frequency *ω* and particle–surface separation *z*_0_ for fixed *k*_D_ *b* = 0.1 (e.g., $z_{0}\approx 9.5$ nm for self-standing graphene with *E*_F_ = 0.3 eV). We assume a lossless resonant scatterer and normalize Γ_SP_(*ω*) to the EELS probability Γ_EELS_(*ω*) in the absence of the 2D layer [Eq. (16)], *ω* to *ω*_D_, and *z*_0_ to the SPP wavelength *λ*_p_. (d) Maximum Γ_SP_(*ω*) along the yellow curve in (c) (left vertical scale) and normalized optimum separation *k*_D_ *z*_0_ (right scale) as a function of frequency.

We consider the interaction between a fast electron and an individual dipolar scatterer placed at the origin (**r** = 0), at a distance *z*_0_ away from a planar surface lying at the *z* = −*z*_0_ plane, as schematically sketched in Figure 1(a). The electron moves parallel to the surface with a velocity $v=v\hat{x}$ at a distance *b* above the scatterer [i.e., the electron trajectory is given by **r**_e_(*t*) = (*vt*, 0, *b*)]. We introduce the optical response of the surface through Fresnel’s reflection coefficients, while the scatterer is described through a frequency-dependent diagonal polarizability tensor $\alpha \left(\omega \right)=\alpha_{\|}\left(\omega \right)\hat{R}⊗\hat{R}+\alpha_{⊥}\left(\omega \right)\hat{z}⊗\hat{z}$ [adopting the notation **R** = (*x*, *y*)]. For the sake of simplicity, we model the scatterer as a point-like particle, which is a good approximation to small scatterers compared to the light wavelength [41]. Using these elements and starting from the field of the electron in free space, we calculate the surface-reflected field and the self-consistent dipole induced at the scatterer. The result is then analyzed in far in-plane regions to quantify the number of polaritons excited by the passage of the electron.

The electron can directly excite SPs (without the presence of a scatterer) under the condition that the line *ω* = *k*_‖_ *v* lies above the mode dispersion curve in *k*_‖_ − *ω* space [i.e., for energies above the crossing point in Figure 1(b)]. While the interplay between direct and particle-assisted SP creation constitutes an interesting scenario for future analysis, we limit the present work to frequencies *ω* below the SP excitation threshold (i.e., direct SP excitation by the electron is kinematically forbidden, but SPs can still be created through the mediation of particle polarization).

### 2.1 Electron field near a planar surface

Working in frequency space *ω*, the electric field directly created by the electron as it moves in free space can be written as [77]

(1) $$\begin{array}{ll} E_{\text{e}}^{\text{dir}}\left(r,\omega \right) & =\frac{ie}{v}\int_{-\infty }^{\infty }\frac{\text{d}k_{y}}{\kappa }\;{\text{e}}^{\text{i}k_{\|}\cdot R-\kappa |z-b|}\\ & \;\times \left(\frac{\omega }{v\gamma^{2}},k_{y},i\;\kappa \;\mathrm{sign}\left\{z-b\right\}\right), \end{array}$$

where $k_{\|}=\left(\omega /v\right)\hat{x}+k_{y}\hat{y}$ is the in-plane wave vector, $\kappa =\sqrt{{\left(\omega /v\gamma \right)}^{2}+k_{y}^{2}}$ describes an evanescent decay away from *z* = *b*, and $\gamma =1/\sqrt{1-v^{2}/c^{2}}$ . This equation shows that the electron field is composed of components satisfying the condition *k*_‖_ ≤ *ω*/*v*, corroborating that modes above the electron line in Figure 1(b) cannot be directly excited by the electron.

The presence of the planar surface at *z* = −*z*_0_ produces a reflected field that can be conveniently expressed in terms of Fresnel’s reflection coefficients $r_{k_{\|}\text{p}}$ and $r_{k_{\|}\text{s}}$ for p and s polarization, respectively. To do so, we note that the integrand in Eq. (1) is orthogonal to the wave vector $k_{\|}+i\;\mathrm{sign}\left\{z-b\right\}\kappa \hat{z}$ (i.e., the field is transverse), so it can be projected on p- and s-polarization components represented by the unit vectors ${\hat{e}}_{k_{\|}\text{p}}^{\pm }=\left(1/kk_{\|}\right)\left(\pm k_{z}k_{\|}-k_{\|}^{2}\hat{z}\right)$ (with *k* = *ω*/*c* and $k_{z}=\sqrt{k^{2}-k_{\|}^{2}+i0^{+}}$ ; here, we take square roots yielding positive real parts, while the infinitesimally small imaginary part is inherited from the adoption of the retarded response formalism to guarantee that Im{*k*_*z*_} > 0) and ${\hat{e}}_{k_{\|}\text{s}}^{\pm }=\left(1/k_{\|}\right)\left(-k_{y}\hat{x}+k_{x}\hat{y}\right)$ , respectively, which we eventually evaluate at *k*_*x*_ = *ω*/*v* and *k*_*z*_ = i*κ*. In these expressions, upper (lower) signs correspond to waves propagating upward (downward). The direct electron field is then expressed in the form

(2a) $$E_{\text{e}}^{\text{dir}}\left(r,\omega \right)=\frac{ek}{v}\overset{\infty }{\underset{-\infty }{\int }}\frac{\text{d}k_{y}}{k_{\|}}\;{\text{e}}^{\text{i}k_{\|}\cdot R-\kappa \left(b-z\right)}$$

$$\;\times \left[-{\hat{e}}_{k_{\|}\text{p}}^{-}+\frac{ik_{y}v}{\kappa \;c}\;{\hat{e}}_{k_{\|}\text{s}}^{-}\right]$$

for *z* < *b* (i.e., it consists of downward waves emanating from the e-beam). By applying the reflection coefficients to each wave vector and polarization component, the reflected field in the *z* > − *z*_0_ region above the surface is found to be

(2b) $$E_{\text{e}}^{\text{ref}}\left(r,\omega \right)=\frac{ek}{v}\overset{\infty }{\underset{-\infty }{\int }}\frac{\text{d}k_{y}}{k_{\|}}\;{\text{e}}^{\text{i}k_{\|}\cdot R-\kappa \left(z+b+2z_{0}\right)}$$

$$\;\times \left[-r_{k_{\|}\text{p}}{\hat{e}}_{k_{\|}\text{p}}^{+}+r_{k_{\|}\text{s}}\frac{ik_{y}v}{\kappa \;c}\;{\hat{e}}_{k_{\|}\text{s}}^{+}\right].$$

The total field generated by the electron in the presence of the surface then becomes $E_{\text{e}}\left(r,\omega \right)=E_{\text{e}}^{\text{dir}}\left(r,\omega \right)+E_{\text{e}}^{\text{ref}}\left(r,\omega \right)$ . Here, we assume *b* > 0 (i.e., the electron moves above the scatterer). An extension to *b* < 0 (electron moving in the region separating the surface from the scatterer) is straightforward but should not add qualitatively different results.

### 2.2 Self-consistent particle-near-surface response

The field generated by the electron polarizes the dipolar particle placed at the origin, which displays a dipole moment

(3) p(ω)=α(ω)⋅E(0,ω)

in the frequency domain. Here, $E\left(0,\omega \right)=E_{\text{e}}\left(0,\omega \right)+E_{\text{dip}}^{\text{ref}}\left(0,\omega \right)$ is the total field acting on the particle, which consists of the electron field and the reflection of the dipole field by the surface $E_{\text{dip}}^{\text{ref}}\left(0,\omega \right)$ .

We follow a similar procedure as with the electron field to express $E_{\text{dip}}^{\text{ref}}\left(0,\omega \right)$ in terms of p- and s-polarization components, starting from the direct dipole field [77] $E_{\text{dip}}^{\text{dir}}\left(r,\omega \right)=\left[k^{2}p\left(\omega \right)+p\left(\omega \right)\cdot \nabla \;\nabla \right]{\text{e}}^{\text{i}kr}/r$ and projecting it on in-plane wave-vector and polarization components to yield [40]

(4a) $$E_{\text{dip}}^{\text{dir}}\left(r,\omega \right)=\frac{ik^{2}}{2\pi }\int \frac{{\text{d}}^{2}k_{\|}}{k_{z}}\;{\text{e}}^{\text{i}k_{\|}\cdot R+\text{i}k_{z}|z|}$$

$$\;\times \underset{\sigma =s,p}{\sum }\left[p\left(\omega \right)\cdot {\hat{e}}_{k_{\|}\sigma }^{s}\right]{\hat{e}}_{k_{\|}\sigma }^{s},$$

where *s* = sign{*z*}. The surface-reflected field is then obtained by introducing the reflection coefficients as

(4b) $$E_{\text{dip}}^{\text{ref}}\left(r,\omega \right)=\frac{ik^{2}}{2\pi }\int \frac{{\text{d}}^{2}k_{\|}}{k_{z}}\;{\text{e}}^{\text{i}k_{\|}\cdot R+\text{i}k_{z}\left(z+2z_{0}\right)}$$

$$\;\times \underset{\sigma =s,p}{\sum }r_{k_{\|}\sigma }\;\left[p\left(\omega \right)\cdot {\hat{e}}_{k_{\|}\sigma }^{-}\right]{\hat{e}}_{k_{\|}\sigma }^{+}.$$

Finally, setting **r** = 0 (the dipole position) and carrying out the azimuthal integral in **k**_‖_, we find

(5) $$E_{\text{dip}}^{\text{ref}}\left(0,\omega \right)=G\left(\omega \right)\cdot p\left(\omega \right)$$

in terms of a Green tensor $G\left(\omega \right)=G_{\|}\left(\omega \right)\hat{R}⊗\hat{R}+G_{⊥}\left(\omega \right)\hat{z}⊗\hat{z}$ with in- and out-of-plane components given by

$$\left[\begin{array}{c} G_{\|}\left(\omega \right)\\ G_{⊥}\left(\omega \right) \end{array}\right]=\frac{i}{2}\int_{0}^{\infty }\frac{k_{\|}\text{d}k_{\|}}{k_{z}}\;{\text{e}}^{2\text{i}k_{z}z_{0}}\;\left[\begin{array}{c} k^{2}\;r_{k_{\|}s}-k_{z}^{2}\;r_{k_{\|}p}\\ 2k_{\|}^{2}\;r_{k_{\|}p} \end{array}\right].$$

Combining this result with Eq. (3), we can write the self-consistently induced dipole as

(6a) $$p\left(\omega \right)=\alpha^{\text{eff}}\left(\omega \right)\cdot E_{\text{e}}\left(0,\omega \right),$$

where

(6b) $$\alpha^{\text{eff}}\left(\omega \right)=\frac{1}{1/\alpha \left(\omega \right)-G\left(\omega \right)}$$

is an effective polarizability that takes into account the effect of the surface, so only the electron field is now required to evaluate the induced dipole [cf. Eqs. (3) and (6a)]. Incidentally, the fraction in Eq. (6b) must be understood as the inverse of the 3 × 3 tensor in the denominator.

### 2.3 The limit of strongly confined surface polaritons

We are interested in generating SPs of small wavelength *λ*_p_ compared with the light wavelength *λ*_0_ = 2*πc*/*ω*. In this limit, we can neglect the contribution of s-polarization components to the surface response (i.e., $r_{k_{\|}\text{s}}\to 0$ ) and approximate *k*_*z*_ ≈ i*k*_‖_ in the dipolar self-interaction (because *k*_‖_ ≫ *ω*/*c*), so the Green tensor reduces to

(7) $$G_{⊥}\left(\omega \right)\approx 2\;G_{\|}\left(\omega \right)\approx \overset{\infty }{\underset{0}{\int }}k_{\|}^{2}\text{d}k_{\|}\;{\text{e}}^{-2k_{\|}z_{0}}\;r_{k_{\|}p}.$$

For simplicity, we take the e-beam to intersect the surface normal at the particle position. Again, we neglect s-polarization components in the surface-reflected electron field, but retain the relativistic *γ* factors, so that our results remain valid at high electron velocities, even though the response of the particle–surface system is treated electrostatically. Then, evaluating Eq. (2b) at the particle position (**r** = 0), we find

(8a) $$E_{\text{e}}^{\text{ref}}\left(0,\omega \right)=-\frac{ie}{v}\int_{-\infty }^{\infty }\text{d}k_{y}\;{\text{e}}^{-\kappa \left(b+2z_{0}\right)}$$

$$\;\times \;r_{k_{\|}\text{p}}\;\left(\kappa \omega /vk_{\|}^{2},0,i\right).$$

For the direct electron field, we can analytically integrate Eq. (1), which yields [41]

(8b) $$E_{\text{e}}^{\text{dir}}\left(0,\omega \right)=\frac{2e\omega }{v^{2}\gamma }\left[\frac{i}{\gamma }K_{0}\left(\frac{\omega b}{v\gamma }\right)\hat{x}+K_{1}\left(\frac{\omega b}{v\gamma }\right)\hat{z}\right],$$

where *K*_*m*_ are modified Bessel functions.

The *λ*_p_ ≪ *λ*_0_ condition is generally satisfied if the surface consists of a thin film, which, neglecting nonlocal and retardation effects, can be described through a frequency-dependent 2D surface conductivity *σ*(*ω*) and treated as a zero-thickness layer. When the film is deposited on a substrate of real permittivity *ϵ*, the Fresnel reflection coefficient for p polarization from the vacuum side reduces to [78]

(9) $$r_{k_{\|}\text{p}}=1+R_{\text{p}}\frac{k_{\text{p}}}{k_{\|}-k_{\text{p}}},$$

where $R_{\text{p}}=1/\bar{\epsilon }$ with $\bar{\epsilon }=\left(\epsilon +1\right)/2$ , while $k_{\text{p}}=i\omega \bar{\epsilon }/2\pi \sigma \left(\omega \right)$ is the complex polariton wave vector identified as a pole in $r_{k_{\|}\text{p}}$ [30], [79], whose existence requires fulfilling the condition Im{*σ*} > 0. The SP wavelength is then defined as *λ*_p_ = 2*π*/Re{*k*_p_}.

We consider long-lived polaritons characterized by low inelastic losses (i.e., Re{*σ*}≪|*σ*|) such as, for example, SPPs in high-quality doped graphene [30], [80], [81], whose surface conductivity can be approximated as

(10) $$\sigma \left(\omega \right)=\frac{e^{2}}{\hbar }\frac{i\omega_{\text{D}}}{\omega +i\gamma }$$

in the Drude model, where *ω*_D_ = *E*_F_/*πℏ* is a frequency weight proportional to the doping Fermi energy *E*_F_ and we introduce a phenomenological inelastic damping rate *γ*. This type of response is also found in thin noble metal films, where $\omega_{\text{D}}=\hbar \omega_{\text{bulk}}^{2}d/4\pi e^{2}$ depends on the bulk plasma energy *ℏω*_bulk_ (e.g., 9.17 eV in silver [82]) and the film thickness *d* [5]. Assuming the surface conductivity in Eq. (10), the SPP dispersion relation obtained from the *k*_‖_ = *k*_p_ pole in Eq. (9) is universally given by $k_{\|}=\left(\bar{\epsilon }\hbar /2\pi e^{2}\omega_{\text{D}}\right)\;\omega \left(\omega +i\gamma \right)$ and exhibits a characteristic $\omega \propto \sqrt{k_{\|}}$ scaling, as shown in Figure 1(b).

Using the reflection coefficient in Eq. (9), the *k*_‖_ integral in Eq. (7) can be performed analytically, leading to

$$G_{⊥}\left(\omega \right)=2\;G_{\|}\left(\omega \right)=\frac{1}{4z_{0}^{3}}+R_{\text{p}}k_{\text{p}}^{3}\left[\frac{1}{\theta }+\frac{1}{\theta^{2}}-{\text{e}}^{-\theta }\;\mathrm{Ei}\left(\theta \right)\right]$$

with *θ* = 2*k*_p_ *z*_0_ and Ei denoting the exponential integral function (see Eqs. 3.353–5 of Ref. [83]). The *ω* dependence of the Green tensor is then encapsulated in *k*_p_. In the low-loss limit (*γ* ≪ *ω*), we can approximate

(11) $$Im\left\{r_{k_{\|}\text{p}}\right\}\approx \pi R_{\text{p}}k_{\text{p}}\delta \left(k_{\|}-k_{\text{p}}\right)$$

with *k*_p_ = 2*π*/*λ*_p_ taken as a real number. Inserting this expression into Eq. (7), we find

(12) $$Im\left\{G_{⊥}\left(\omega \right)\right\}=2\;G_{\|}\left(\omega \right)=\pi R_{\text{p}}k_{\text{p}}^{3}{\text{e}}^{-2k_{\text{p}}z_{0}}.$$

We use this result in what follows.

In the low-loss limit, noticing that the polariton pole dominates the surface response, we can also obtain an analytical expression for the surface-reflected electron field by calculating the residue due to the *k*_‖_ = *k*_p_ pole of $r_{k_{\|}\text{p}}$ in Eq. (8a). To this end, we multiply and divide the $R_{\text{p}}$ term in the integrand by *k*_‖_ − *k*_p_, close the integration contour in the complex *k*_*y*_ plane, and ignore the contributions of branching points and any other poles in the integrand. The field contributed by the first term in the right-hand side of Eq. (9) takes the same form as the direct field in Eq. (8b), but with *b* replaced by *b* + 2*z*_0_ and the sign of the *x* component flipped. In this polariton-pole approximation (PPA), inserting the result in Eq. (6a) together with the direct field from Eq. (8b), the electron field in the absence of the particle reduces to

(13) $$\begin{array}{ll} E_{\text{e}}\left(0,\omega \right) & \approx \frac{2e\omega }{v^{2}}\left[\left\{\frac{i}{\gamma^{2}}K_{0}\left[\frac{\omega b}{v\gamma }\right]-\frac{i}{\gamma^{2}}K_{0}\left[\frac{\omega \left(b+2z_{0}\right)}{v\gamma }\right]\right.\right.\\ & \;\left.+\frac{\pi R_{\text{p}}\nu \;{\text{e}}^{-\nu \omega \left(b+2z_{0}\right)/v}}{\sqrt{\mu^{2}-1}}\right\}\hat{x},\\ & \;+\left\{\frac{1}{\gamma }K_{1}\left[\frac{\omega b}{v\gamma }\right]+\frac{1}{\gamma }K_{1}\left[\frac{\omega \left(b+2z_{0}\right)}{v\gamma }\right]\right.\\ & \left.\left.\;+\frac{i\pi R_{\text{p}}\mu^{2}{\text{e}}^{-\nu \omega \left(b+2z_{0}\right)/v}}{\sqrt{\mu^{2}-1}}\right\}\hat{z}\right], \end{array}$$

where *μ* = *k*_p_ *v*/*ω* and $\nu =\sqrt{\mu^{2}-v^{2}/c^{2}}$ . The first, second, and third terms inside each of the curly brackets in Eq. (13) origine from the direct electron field [Eq. (8b)], the surface-reflected field contributed by the unity term in $r_{k_{\|}\text{p}}$ , and the result of the remaining part of $r_{k_{\|}\text{p}}$ , respectively.

### 2.4 Polariton excitation probability

Under the conditions of Figure 1(a) and (b), assuming that the electron line *ω* = *k*_‖_ *v* lies below the mode dispersion curve, direct SP excitation by the electron is kinematically forbidden, so SPs are exclusively excited *via* the induced particle dipole [Eq. (6a)]. We now calculate the number of the so-emitted surface quanta. To this end, we first consider a dipole **p**(*t*) placed in free space. By integrating the radial Poynting vector over both time and the surface of a large sphere centered at the dipole, we find a total emitted energy $\left(2/3\pi c^{3}\right)\int_{0}^{\infty }\omega^{4}\text{d}\omega \;|p\left(\omega \right){|}^{2}$ . We then divide each frequency component by *ℏω* to obtain the total number of emitted quanta $\int_{0}^{\infty }\text{d}\omega \;\Gamma_{0}\left(\omega \right)$ , where Γ_0_(*ω*) = (2*ω* ^3^/3*πℏc* ^3^)|**p**(*ω*)|^2^ gives the spectral decomposition of the emission probability. This result differs by a factor of 1/2*π* from the decay rate of a transition dipole in free space [84].

For clarity, we note that the time-domain induced dipole is obtained from Eq. (6a) by performing the Fourier transform **p**(*t*) = (2*π*)^−1^ ∫d*ω* e^−i*ωt*^ **p**(*ω*). Now, following the same analysis as in Ref. [84], we find that the presence of a material structure changes the spectral probability to Γ(*ω*) = Γ_0_(*ω*) + (1/*πℏ*) Im{p*(*ω*) ⋅**E** ^ind^}, where **E** ^ind^ is the self-induced electric field at the dipole position. In particular, near a planar surface, this field is given by $E_{\text{dip}}^{\text{surf}}\left(0,\omega \right)$ in Eq. (5), and consequently, $\Gamma \left(\omega \right)=\Gamma_{0}\left(\omega \right)+\left(1/\pi \hbar \right)\;Im\left\{|p_{\|}\left(\omega \right){|}^{2}G_{\|}\left(\omega \right)+|p_{z}\left(\omega \right){|}^{2}G_{⊥}\left(\omega \right)\right\}$ . Adopting again the electrostatic limit [Eq. (7)], this leads to

(14) $$\Gamma_{\text{SP}}\left(\omega \right)=\frac{1}{\pi \hbar }\;\left[|p_{\|}\left(\omega \right){|}^{2}+2|p_{z}\left(\omega \right){|}^{2}\right]\;Im\left\{G_{\|}\left(\omega \right)\right\}$$

for the spectrally resolved probability of emitting SPs, where $Im\left\{G_{\|}\left(\omega \right)\right\}$ is proportional to $R_{\text{p}}$ , indicating that Γ_SP_(*ω*) is only contributed by SP emission within the geometry and approximations under consideration. Incidentally, this result coincides with the integral of the absorption density within the 2D film (see Section 5.3 in Methods).

We aim to maximize the probability of exciting surface modes, which is enhanced when *α* ^eff^(*ω*) increases [see Eq. (6a)], and therefore, optimum emission should occur if the scatterer is lossless (i.e., composed of nonabsorbing materials, such that Im{−1/*α*_*s*_(*ω*)} = 2*k* ^3^/3 for each of the polarization directions *s* = ‖, ⊥) and resonant (i.e., $\text{Re}\left\{1/\alpha_{s}\left(\omega \right)-G_{s}\left(\omega \right)\right\}=0$ , a condition that can be generally fulfilled at specific resonance frequencies [40]). Combining these two conditions, we have

(15) $$\alpha_{s}^{\text{eff}}\left(\omega \right)=\frac{i}{2k^{3}/3+\text{Im}\left\{G_{s}\left(\omega \right)\right\}}$$

for the effective polarizability of a perfect scatterer.

To illustrate the magnitude of Γ_SP_(*ω*) in Eq. (14) and its dependence on geometrical parameters, we find it convenient to normalize it to the EELS probability produced by a free-standing particle (without the surface) [85] $\Gamma_{\text{EELS}}\left(\omega \right)=\left(4e^{2}\omega^{2}/\pi \hbar v^{4}\gamma^{2}\right)\left[\left(1/\gamma^{2}\right),K_{0}^{2},\left(\omega b/v\gamma \right),\text{Im},\left\{\alpha_{\|}\right\},+,K_{1}^{2},\left(\omega b/v\gamma \right),\text{Im}\right.\left.\left\{\alpha_{⊥}\right\}\right]$ , which we evaluate for a perfect scatterer by setting *α*(*ω*) = 3i/2*k* ^3^:

(16) $$\Gamma_{\text{EELS}}\left(\omega \right)=\frac{6\;e^{2}c^{3}}{\pi \hbar v^{4}\gamma^{2}\omega }\left[\frac{1}{\gamma^{2}}K_{0}^{2}\left(\frac{\omega b}{v\gamma }\right)+K_{1}^{2}\left(\frac{\omega b}{v\gamma }\right)\right].$$

The resulting ratio Γ_SP_(*ω*)/Γ_EELS_(*ω*) is presented in Figure 1(c) as a function of frequency and particle–surface separation *z*_0_ for a fixed impact parameter *b*, assuming a lossless resonant particle [Eq. (15)] and a 2D Drude material described by the conductivity of Eq. (10).

Despite the indirect mechanism for SP generation, the probability plotted in Figure 1(c) takes sizeable values about one order of magnitude lower than the maximum possible EELS probability for a dipolar scatterer. This figure also reveals the presence of a maximum in the emission probability Γ_SP_(*ω*) for an optimum value of *z*_0_. The latter depends on SP frequency and lies in the *z*_0_ < *λ*_p_ range [Figure 1(c), yellow line]. As a function of SP frequency, this maximum remains nearly constant over the explored frequency range [Figure 1(d)] for the particular choice of the chosen parameters. We note that Figure 1(b) and (d) is calculated analytically using Eqs. (6a), (12), (14), and (15) combined with the PPA expression for the electron field in Eq. (13), but almost identical results are obtained when the latter is computed without approximations from the sum of Eqs. (8a) and (8b) (Supplementary Materials, Figure S1).

Upon inspection of the analytical expressions in Eqs. (12) and (13), we find that Im{G(ω)} and the PPA electron field **E**_e_(0, *ω*) depend on *ω* and *z*_0_ only through the ratios *ω*/*ω*_D_ and *z*_0_/*λ*_p_, and consequently, the PPA approach yields universal results for a lossless resonant scatterer and a thin film described through a low-loss Drude conductivity [Eq. (10)]. The SP emission probability is also dependent on damping and impact parameter through *γ*/*ω*_D_ and *b*/*λ*_p_. To corroborate this result, we show that the emission probabilities obtained for graphene and a thin silver film (also described in the Drude model as explained in Section 5.1; see Methods) yield nearly identical results as a function of *ω*/*ω*_D_ and *z*_0_/*λ*_p_ (Supplementary Materials, Figure S2).

### 2.5 Excitation of graphene plasmons assisted by a hBN disk

So far, we have considered lossless, resonant scatterers to mediate the excitation of SPPs in Drude-like films. We now show that similar results are obtained when these ideal conditions are relaxed and we consider resonant hBN particles to excite mid-infrared plasmons in graphene. These particles have low losses, although still much higher than those associated with radiative emission in free space (see Supplementary Materials, Figure S4). However, losses arising from SP emission when the particles are placed close to the surface become dominant at short separations, so the small intrinsic damping due to material losses in the particle does not play a major effect.

**Figure 2:**
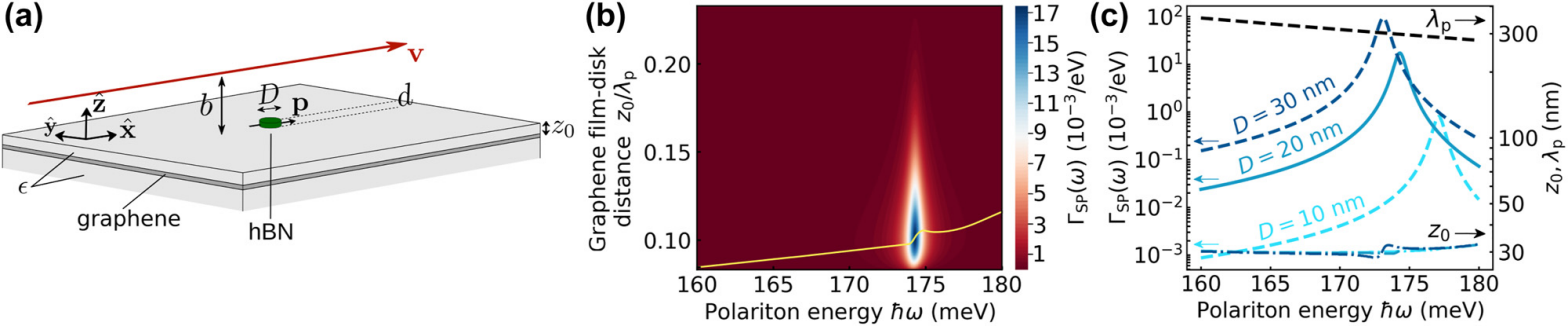
Free-electron excitation of graphene plasmons mediated by a hBN disk scatterer. (a) Geometry under consideration, comprising a graphene film and an hBN disk of diameter *D* and thickness *d*. The disk is directly deposited on the top dielectric layer of thickness *z*_0_. The electron passes at a distance *b* above the disk. The dielectric substrate and coating layer have a permittivity *ϵ*. (b) Spectrally resolved probability of graphene-plasmon emission Γ_SP_(*ω*) under the conditions of (a) as a function of plasmon energy *ℏω* and coating-layer thickness *z*_0_ for *D* = 20 nm, *d* = 1 nm, *ϵ* = 2, *b* = 10 nm, and *v* = 0.04 *c*. We set the graphene Fermi energy to *E*_F_ = 1 eV. (c) Maximum Γ_SP_(*ω*) (left vertical scale, solid curve) and optimum coating thickness *z*_0_ along with the SP wavelength *λ*_p_ (right scale) as a function of emission frequency under the conditions of (b). In (c), we also show results for the maximum Γ_SP_(*ω*) with *D* = 10 nm and 30 nm (see labels).

We then consider a feasible configuration in which a hBN disk (thickness *d* and diameter *D* ≫ *d*) acts as a relatively lossless scatterer that couples to SPs in a graphene film. To make the system more realistic, we assume the graphene film to be supported on a dielectric substrate (permittivity *ϵ*) and coated by a dielectric film of thickness *z*_0_ (same material). The hBN disk is taken to be directly deposited on the surface of the coating layer, with vacuum above it [Figure 2(a)]. Again, we assume that all the involved distances are small compared with the light wavelength, so the system can be described in the electrostatic limit. In addition, the thickness of the hBN disk is assumed to be small compared with the polariton wavelength, and consequently, it can be represented by a surface conductivity (see Section 5.1 in Methods).

The effective disk polarizability cannot be calculated by following the approach used above for a self-standing scatterer because the Green tensor diverges as the disk-surface separation is reduced. Instead, we use an analytical expression for the polarizability of a disk deposited on a homogeneous substrate obtained from an electrostatic modal expansion [86], and then introduce the effect of the graphene layer through an *ad hoc* Green tensor, as shown in Methods (Section 5.2). In addition, the surface reflection coefficient needs to be modified to account for the new layered structure. A Fabry–Perot-type of analysis readily leads to

(17) $$r_{k_{\|}\text{p}}=\frac{r_{0}+{\text{e}}^{-2k_{\|}z_{0}}}{1+r_{0}r_{k_{\|}\text{p}}^{\prime }{\text{e}}^{-2k_{\|}z_{0}}},$$

where *r*_0_ = (*ϵ* − 1)/(*ϵ* + 1) and $r_{k_{\|}\text{p}}^{\prime }=k_{\|}/\left(k_{\|}-k_{\text{p}}^{\prime }\right)$ with $k_{\text{p}}^{\prime }=\text{i}\omega \epsilon /2\pi \sigma \left(\omega \right)$ are the reflection coefficients of the homogeneous *ϵ* surface and the *ϵ*-embedded graphene layer [78], respectively.

Finally, the SP emission probability differs for the geometry in Figure 2(a) relative to Figure 1(a). A calculation based on the absorption density within the graphene film yields the result (see Section 5.3 in Methods)

(18) $$\Gamma_{\text{SP}}\left(\omega \right)=\frac{1}{\pi \hbar }\;|p_{x}\left(\omega \right){|}^{2}\;Im\left\{G_{\|}\left(\omega \right)\right\},$$

similar to Eq. (14) but with *p*_*z*_ = 0 (i.e., the induced disk dipole is parallel to the surface) and a modified Green tensor

(19) $$\begin{array}{ll} Im\left\{G_{\|}\left(\omega \right)\right\} & \approx {\left(\frac{2\epsilon }{\epsilon +1}\right)}^{2}\\ & \;\times \overset{\infty }{\underset{0}{\int }}k_{\|}^{2}\text{d}k_{\|}\;{\text{e}}^{-2k_{\|}z_{0}}\;\frac{Im\left\{r_{k_{\|}p}^{\prime }\right\}}{{\left|1+r_{0}r_{k_{\|}\text{p}}^{\prime }{\text{e}}^{-2k_{\|}z_{0}}\right|}^{2}}, \end{array}$$

which reduces to Eq. (7) when setting *ϵ* = 1.

We now use Eq. (18) combined with Eq. (19) to calculate the surface-polariton emission probability in the configuration of Figure 2(a). Here, the induced dipole $p_{x}\left(\omega \right)=\alpha_{\|}^{\text{eff}}\left(\omega \right)E_{\text{e},x}\left(0,\omega \right)$ is obtained by using the modified polarizability in Eqs. (25) (see Section 5.2 in Methods) together with the electron field given by the sum of Eqs. (8a) and (8b). In particular, Eq. (8a) is evaluated by inserting the multilayer reflection coefficient given in Eq. (17) with *z*_0_ = 0 because the electron moves at a distance *b* from the outer surface on which the direct electron field is reflected.

In Figure 2(b), we plot the resulting probability Γ_SP_(*ω*) as a function of disk–2D-layer spacer *z*_0_ and frequency around the upper hBN Reststrahlen band for a disk thickness *d* = 1 nm (corresponding to 3 monolayers) and diameter *D* = 20 nm ≫ *d*. This choice for the disk diameter is motivated to enable a dipolar resonance within that band and boost the polarizability. In agreement with Figure 1, coupling to graphene plasmons is also maximized for an optimum frequency-dependent value of *z*_0_/*λ*_p_. However, we now observe a strong dependence of Γ_SP_(*ω*) on mode frequency, with a sharp increase at a specific spectral position that is controlled by the intrinsic diameter-dependent resonance of the disk [see Figure 2(b) for *D* = 20 nm and Figure 2(c) for *D* = 10, 10, and 30 nm; see also Supplementary Materials, Figure S3(a) for the disk resonance frequency]. This feature demonstrates that the coupling scheme here considered allows us to select the excitation frequency of narrowband plasmons in a region where they would otherwise be inaccessible via direct electron excitation. Interestingly, the optimum *z*_0_/*λ*_p_ ratio takes smaller values than in Figure 1, presumably as a result of the weak scattering strength of the hBN disk compared to a perfect scatterer (see Supplementary Materials, Figure S4). This hypothesis is consistent with the observation that the maximum Γ_SP_(*ω*) and the optimum *z*_0_ are both increasing with disk diameter [see the probability curves for *D* = 10, 20, and 30 nm in Figure 2(c)].

## 3 Polaritonic Smith–Purcell emission

**Figure 3:**
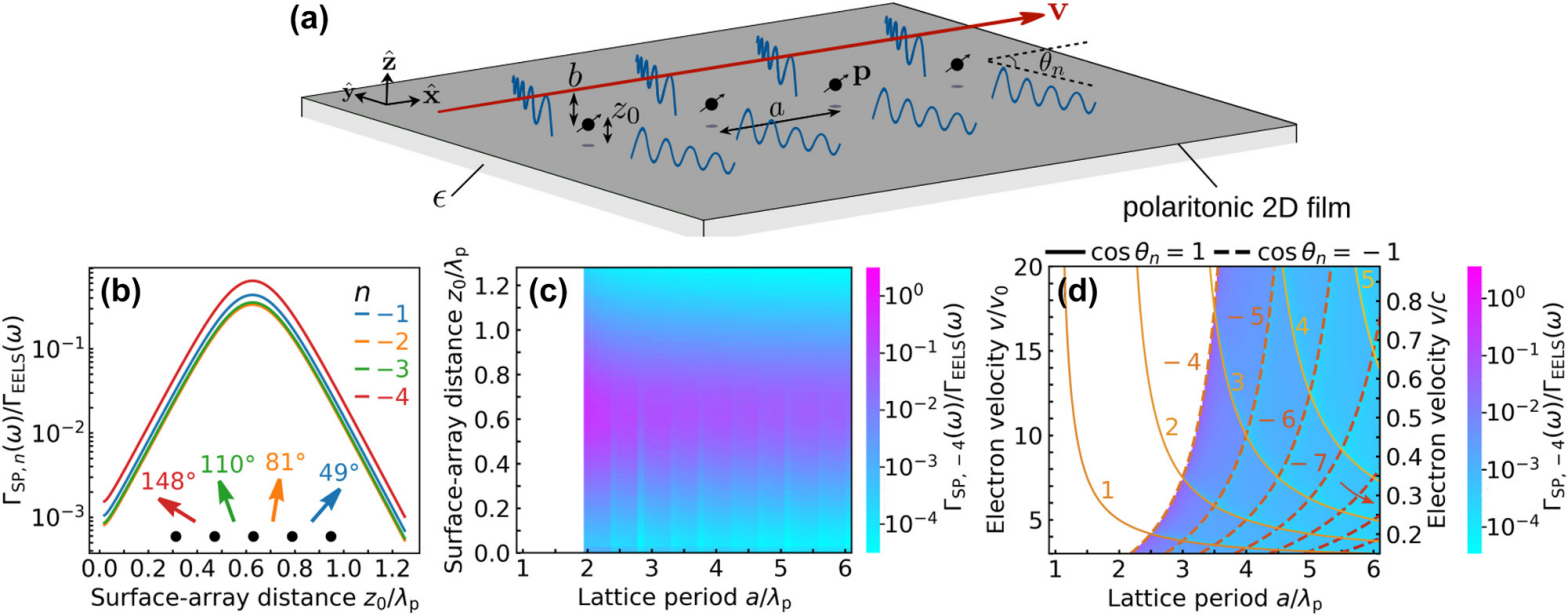
Polaritonic Smith–Purcell effect. (a) Scheme with the geometry under consideration, similar to Figure 1(a) but with a linear array of particles spaced by a period *a* along the direction of electron motion. The in-plane emission angle *θ*_*n*_ is indicated for one of the orders *n* [see Eq. (20)]. (b) Probability of SPP emission Γ_SP,*n*_(*ω*) for different orders *n* normalized per particle and divided by the self-standing-particle EELS probability Γ_EELS_(*ω*) [Eq. (16)] as a function of scaled array–surface distance *z*_0_/*λ*_p_ for fixed frequency *ω* = 0.4 *ω*_D_. We assume perfect scatterers, a Drude film like that in Figure 1, and parameters *a*/*λ*_p_ = 2, *v* = 2.2 *v*_0_ ≈ 0.2 *c*/(*ϵ* + 1), and *k*_D_ *b* = 0.1. The corresponding emission angles *θ*_*n*_ are indicated in the inset (top-view scheme). (c, d) Ratio Γ_SP,*n*=−4_(*ω*)/Γ_EELS_(*ω*) as a function of either (c) *a*/*λ*_p_ and *z*_0_/*λ*_p_ for *v*/*v*_0_ = 2.2, or (d) *a*/*λ*_p_ and *v*/*v*_0_ for *z*_0_/*λ*_p_ = 0.6, with all other parameters the same as in (b). The right vertical scale in (d) shows *v*/*c* for *ϵ* = 1. White regions in (c, d) correspond to the condition that the *n* = −4 Smith–Purcell emission is kinematically forbidden. Probability minima contours are observed as light-blue regions in (d) and marked by solid and dashed curves corresponding to the conditions cos *θ*_*n*_ = 1 and −1, respectively, for the values of *n* indicated by labels.

Smith–Purcell radiation emission [76] occurs when an e-beam is oriented parallel to a grating such that there is a coherent superposition in the far-field emission associated with periodic elements. A “simple Huygens construction” [76] shows that broadband light is emitted along wavelength-dependent directions satisfying the phase-matching relation cos *θ*_*n*_ = *nλ*_0_/*a* + *c*/*v*, where *a* is the period and *θ*_*n*_ is the emission angle relative to the electron velocity vector $v=v\hat{x}$ when consecutive grooves contribute with a relative time delay given by an integral number *n* of optical periods [41]. Here, we extend this concept to the emission of polaritons by generalizing the results of Section 2 to a linear array of scatterers, as depicted in Figure 3(a). From the Huygens construction sketched in the figure, we find that the SP emission produced by an electron running parallel to the array is subject to the modified phase-matching condition

(20) $$\cos\theta_{n}=\lambda_{\text{p}}\left(\frac{n}{a}+\frac{c}{v\lambda_{0}}\right).$$

Incidentally, while *n* < 0 is required by the traditional version of this effect to obtain a real emission angle *θ*_*n*_ for free photons, polaritonic emission with a positive *n* is also possible for electrons moving faster than the SP phase velocity (i.e., *v* > *c λ*_p_/*λ*_0_) and *λ*_p_ < *a*. Here, we consider parameters for which direct excitation of polaritons (coincident with *n* = 0 emission) is kinematically forbidden.

### 3.1 Electron interaction with a linear array of scatterers

To analyze polaritonic Smith–Purcell emission, we assume again low inelastic losses, so that SPs can propagate far from the interaction region, where the direct and surface-reflected electron fields are negligible at frequencies below the crossing of the electron line and the mode dispersion [see Figure 1(b)]. In those far surface regions, the polariton field is dominated by the contribution of the *k*_‖_ = *k*_p_ pole in $r_{k_{\|}\text{p}}$ [Eq. (9)] to $E_{\text{dip}}^{\text{ref}}\left(r,\omega \right)$ [Eq. (4b)]. In the electrostatic limit, this contribution to the field reduces to $E_{\text{dip}}^{\text{ref}}\left(r,\omega \right)=-\nabla \phi_{\text{dip}}^{\text{ref}}\left(r,\omega \right)$ , written in terms of the associated scalar potential

(21) $$\phi_{\text{dip}}^{\text{ref}}\left(r,\omega \right)=\int \frac{{\text{d}}^{2}k_{\|}}{{\left(2\pi \right)}^{2}}\;{\text{e}}^{\text{i}k_{\|}\cdot R}\;{\tilde{\phi }}_{\text{dip}}^{\text{ref}}\left(k_{\|},z,\omega \right)$$

with

$${\tilde{\phi }}_{\text{dip}}^{\text{ref}}\left(k_{\|},z,\omega \right)=2\pi \;{\text{e}}^{-k_{\|}\left(z+2z_{0}\right)}\;r_{k_{\|}\sigma }\;p\left(\omega \right)\cdot \left(i{\hat{k}}_{\|}+\hat{z}\right)$$

for an individual dipole placed at the origin.

We now consider an infinite linear array of period *a* formed by dipolar scatterers at positions $R_{j}=ja\hat{x}$ , indexed by an integer number *j*. Because of periodicity, the external electron potential at a generic scatterer *j* ≠ 0 differs from the potential at scatterer *j* = 0 by just a phase factor e^i(*ω*/*v*)*aj*^ [see Eq. (1)], and this dependence is directly imprinted on the self-consistent dipole fields. Therefore, the potential produced by all dipoles in the array can be written as $\phi_{\text{array}}^{\text{ref}}\left(r,\omega \right)=\sum_{j=-\infty }^{\infty }{\text{e}}^{\text{i}\left(\omega /v\right)aj}\phi_{\text{dip}}^{\text{ref}}\left(r-R_{j},\omega \right)$ in terms of the potential of one dipole at *j* = 0 [Eq. (21)]. We can perform the *j* sum by using the identity $\sum_{j=-\infty }^{\infty }{\text{e}}^{\text{i}\left(\omega /v-k_{x}\right)aj}=\left(2\pi /a\right)\sum_{n=-\infty }^{\infty }\delta \left(k_{x}-k_{nx}\right)$ , where *k*_*nx*_ = *ω*/*v* + 2*πn*/*a* with *n* running over diffraction orders. The *δ*-functions in this expression allow us to readily carry out the *k*_*x*_ integral and find the result

(22) $$\phi_{\text{array}}^{\text{ref}}\left(r,\omega \right)=\frac{1}{2\pi a}\overset{\infty }{\underset{n=-\infty }{\sum }}\int \text{d}k_{y}\;{\text{e}}^{\text{i}k_{n\|}\cdot R}\;{\tilde{\phi }}_{\text{dip}}^{\text{ref}}\left(k_{n\|},z,\omega \right)$$

with $k_{n\|}=k_{nx}\hat{x}+k_{y}\hat{y}$ .

At large transverse distances |*y*| from the array, we expect the potential to be dominated by SP components emanating from the *k*_‖_ = *k*_p_ pole in Eq. (9). Using the same PPA procedure that allowed us to perform the *k*_*y*_ integral in Eq. (8a) and obtain Eq. (13), we transform Eq. (22) into

(23) $$\begin{array}{ll} \phi_{\text{array}}^{\text{ref}}\left(r,\omega \right) & \approx \frac{2\pi i\;R_{\text{p}}k_{\text{p}}^{2}}{a}\;p\left(\omega \right)\cdot \left(i{\hat{k}}_{n\|}+\hat{z}\right)\\ & \;\times \sum_{n=-\infty }^{\infty }\frac{1}{k_{ny}}\;{\text{e}}^{\text{i}k_{n\|}\cdot R-k_{\text{p}}\left(z+2z_{0}\right)}, \end{array}$$

with $k_{y}\to k_{ny}=\sqrt{k_{\text{p}}^{2}-k_{nx}^{2}}$ . The far in-plane field is then found to be made of different diffracted SP plane waves labeled by *n*. The associated wave vectors **k**_*n*‖_ form angles *θ*_*n*_ with the *x* axis (i.e., the e-beam) determined by cos *θ*_*n*_ = *k*_*nx*_/*k*_p_. This condition can directly be recast into Eq. (20) by neglecting the imaginary part of the SP wave vector and writing *k*_p_ = 2*π*/*λ*_p_.

Periodicity also allows us to write the induced dipoles as **p**(*ω*) e^i(*ω*/*v*)*aj*^, where the dependence on the position of each particle *j* reduces to a phase factor. In the absence of interaction among particles, **p**(*ω*) would be given by Eqs. (6a) and (6b). However, particle–particle interaction may become relevant for strong scatterers and small separations, so we include it in our calculations by defining the effective polarizability of each particle in the array as in Eq. (6b) but with the Green tensor supplemented by the contribution of the direct and surface-reflected fields produced by the rest of the dipoles. This is calculated in Methods (see Section 5.4), and in what follows, we use Eq. (6b) combined with Eq. (29) to obtain *α* ^eff^(*ω*) for particles in the array.

To obtain the number of SPs produced by the passage of the electron, we consider the power density transported by a mode characterized by an electrostatic electric field $E_{0}\left(\hat{x}+i\hat{z}\right)\;{\text{e}}^{k_{\|}\left(\text{i}x-z-z_{0}\right)-\text{i}\omega t}+c.c.$ in the vacuum region outside (*z* > − *z*_0_) a polariton-supporting planar surface placed at the *z* = −*z*_0_ plane. The power per unit of length along the transverse direction *y* is given by [39] $I_{\text{p}}=|\omega E_{0}^{2}/\left(2\pi R_{\text{p}}k_{\text{p}}^{2}\right)|$ , where we assume a reflection coefficient $r_{k_{\|}\text{p}}\approx R_{\text{p}}k_{\text{p}}/\left(k_{\|}-k_{\text{p}}\right)$ dominated by a pole at *k*_‖_ = *k*_p_ and neglect inelastic losses (i.e., Im{*k*_p_}≪|*k*_p_|, so *k*_p_ is treated as a real number). We now substitute *E*_0_ by the in-plane field amplitude associated with the *n*th diffracted beam in Eq. (23) [i.e., $2\pi |R_{\text{p}}k_{\text{p}}^{2}\;\left(ip_{x}\cos\theta_{n}+p_{z}\right)|/a\sin\theta_{n}$ , where we have used the relation *k*_*ny*_ = *k*_p_ sin *θ*_*n*_], then divide by *ℏω* to transform energy into number of SP quanta, and finally multiply by the length *a* sin *θ*_*n*_ (the period projected on the SP wavefront direction). By following this procedure, we obtain the expression

(24) $$\Gamma_{\text{SP},n}\left(\omega \right)=\frac{8\pi^{3}|R_{\text{p}}|\;|i\;p_{x}\left(\omega \right)\cos\theta_{n}+p_{z}\left(\omega \right){|}^{2}}{\hbar \;\lambda_{\text{p}}^{2}\;a\sin\theta_{n}}$$

for the number of emitted SPs normalized to the number of scatterers in the array. For a given set of geometrical parameters and frequency, Smith–Purcell emission is only allowed for orders *n* satisfying the condition −*k*_p_ ≤ 2*πn*/*a* + *ω*/*v* ≤ *k*_p_. In addition, Eq. (24) suggests that the Smith–Purcell emission at a given order *n* is boosted when polaritons are emitted along the direction of the array (i.e., sin *θ*_*n*_ = 0), as we corroborate below.

We consider an array of perfect scatterers like the one sketched in Figure 3(a) and evaluate Γ_SP,*n*_(*ω*) using Eq. (24) with **p**(*ω*) calculated from Eqs. (6a), (13) and (15). The latter involves *s* = *xx* and *zz* components evaluated from Eq. (29) (notice that the direct lattice sum does not contribute to the imaginary part of the Green tensor). An illustrative result is presented in Figure 3(b) for all allowed emission orders *n*, normalized to the perfect-scatterer EELS probability [Eq. (16)]. Like in the configuration studied in Figure 1 for an individual scatterer, we find again a maximum emission at an optimum array–surface separation *z*_0_, which is roughly independent of the order *n*. In Figure 3(c), we show the dependence of Γ_SP,*n*_ for the strongest allowed mode *n* = −4 as a function of lattice period *a* and surface–array distance *z*_0_. We observe singularities in the probability at specific values of *a* corresponding to the onset of new emission orders *n* < −1 (see Supplementary Materials, Figure S5). Nevertheless, the optimum value of *z*_0_ that maximizes the emission is roughly independent of *a*. Incidentally, *n* = −4 emission is kinematically forbidden in the white regions in Figure 3(c) and (d). For each emission order *n*, plasmonic Smith–Purcell emission can thus be finely tuned by playing with the electron velocity, the period, and the array–surface separation.

Interestingly, similar singularities of Γ_SP,−4_ as a function of period *a* and electron velocity *v* are present in Figure 3(d), corresponding to light-blue contours in the color plot. In particular, contours with a positive slope emerge from the condition *θ*_*n*_ = *π* (i.e., anti-parallel emission relative to the electron velocity vector) for different orders *n* = −4, − 5, … (from left to right). The origin of these features is analogous to lattice resonances in particle arrays [87], emerging when the wavelength matches the lattice period, or equivalently, at the onset of every diffraction order. Ultimately, this is the result of the accumulation of in-phase fields emanating from the different particle dipoles in the array [88]. In our system, the array period is small compared with the light wavelength, so free light propagation cannot produce in-phase contributions. However, fields propagating as surface modes can be in phase when the polariton wavelength *λ*_p_ is close to the period *a*. This translates into a divergent-like behavior of the lattice sum in Eq. (28) (see Supplementary Materials, Figure S5 for a plot of this sum), that in turn permeates the induced dipoles and the resulting emission probability in Eq. (24). Likewise, features in Figure 3(d) emerging as negative-slope contours also originate in lattice resonances for forward-propagating polaritons at *θ*_*n*_ = 0 with *n* = 1, 2, … from left to right.

### 3.2 Smith–Purcell emission with an array of hBN nanodisks

**Figure 4:**
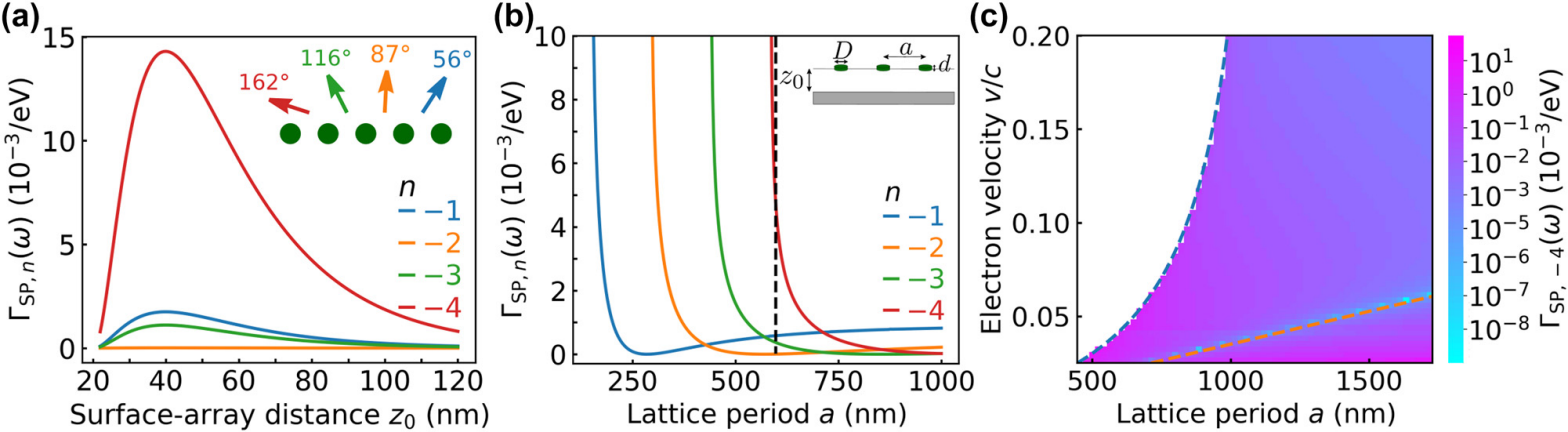
Smith–Purcell emission of surface-plasmon polaritons in grapheme. We consider a geometry similar to Figure 2(a) but with an array of hBN disks instead of just an individual particle and setting *ϵ* = 1 for simplicity [see inset in (b)]. (a) Emission probability per particle Γ_SP,*n*_(*ω*) for different orders *n* and fixed energy *ℏω* = 174 meV (SP wavelength *λ*_p_ = 299 nm) as a function of *z*_0_. We set the period to *a* = 2 *λ*_p_ = 598 nm, while the rest of the disk and substrate parameters are the same as in Figure 2. Emission angles *θ*_*n*_ for the propagating orders *n* are indicated in the inset on a top view of the linear array (green circles). (b) Emission probability per particle for orders shown in (a) as a function of period *a* for *z*_0_ = 40 nm and *v* = 0.04 *c*. The dashed-black line marks the value *a* = 598 nm used in panel (a). Each order *n* exhibits a vertical asymptote at a specific value of *a,* below which is not allowed. (c) Emission probability per particle for *n* = −4 as a function of *a* and *v*/*c* for *z*_0_ = 40 nm, with all other parameters the same as in (b). The white region corresponds to conditions for which *n* = −4 Smith–Purcell emission is kinematically forbidden. The conditions cos *θ*_−4_ = −1 and cos *θ*_−4_ = 0 are indicated by dashed-blue and dashed-orange curves, respectively.

In Figure 4, we consider a linear array of hBN nanodisks with similar substrate and size parameters as in Figure 2. For simplicity, we assume *ϵ* = 1 (this condition is nearly met by amorphous silica at the mid-infrared frequencies under consideration), so we can apply the above formalism and calculate the probability from Eq. (24) with $p_{x}\left(\omega \right)=\alpha_{xx}^{\text{eff}}\left(\omega \right)E_{\text{e},x}\left(0,\omega \right)$ and *p*_*z*_(*ω*) = 0. Here, we use Eq. (8) for *E*_e,*x*_(0, *ω*) with the reflection coefficient $r_{k_{\|}p}$ taken from Eq. (17); we set $\alpha_{xx}^{\text{eff}}\left(\omega \right)={\left[\alpha_{xx}\left(\omega \right)-G_{xx}\left(\omega \right)\right]}^{-1}$ with *α*_*xx*_(*ω*) defined by Eq. (25a); and $G_{xx}\left(\omega \right)$ is computed by numerical integration of Eq. (28) (see Section 5.4 in Methods). We note that $\bar{\epsilon }=R_{\text{p}}=1$ because of the choice of *ϵ* = 1.

Figure 4(a) shows the Smith–Purcell emission probability for all allowed orders. Similar to Figure 3, the Smith–Purcell emission probability is maximized at an optimum value of *z*_0_ that is rather independent of *n*. This effect can be understood for the disks under consideration because there is no out-of-plane polarization (i.e., *p*_*z*_ = 0), and therefore, the dependences of Eq. (24) on either *n* or *z*_0_ can be factorized. Interestingly, the emission is strongest for *n* = −4 because the chosen lattice period *a* is close to the onset of this order [see Figure 4(b)], so the emission angle *θ*_−4_ = 162° is close to grazing [see inset of Figure 4(a)] and the probability scales as 1/sin *θ*_−4_ [see Eq. (24)]. In Figure 4(c), we show the onset of the *n* = −4 order by a dashed-blue curve corresponding to the condition *θ*_−4_ = 180°, which makes Γ_SP,−4_ divergent. Conversely, cos *θ*_−4_ = 90° and Γ_SP,−4_ vanishes along the dashed-orange line [see also Eq. (24)].

## 4 Conclusions

In summary, we show that free electrons can efficiently excite SPs assisted by the mediation of scatterers placed in the vicinity of a polariton-supporting surface. Our semi-analytical theory reveals that the emission probability is maximized for lossless resonant scatterers placed at an optimum distance from the surface. This method enables the emission of surface polaritons even if the electron cannot directly excite polaritons due to kinematic constraints. In particular, polaritons of relatively large phase velocity can still be excited by low-energy electrons. We further explore polaritonic Smith–Purcell emission mediated by linear arrays of scatterers under excitation by electrons moving parallel to the array. An optimum array–surface separation is also observed for maximum emission. Interestingly, lattice resonances emerge as singularities in the Smith–Purcell excitation probability, signaled by the onset of new emission orders when their corresponding polariton wavelengths match the period of the array. The fact that each Smith–Purcell emission order emits preferentially at a given angle that can be controlled by the properties of the surface, scatterer, and electron beam provides additional knobs to manipulate the excitation and steering of polaritons in extended surfaces without requiring their patterning. Similarly, the engineering of such arrays of scatterers (for example, through their individual polarizabilities or positions) could enable further control of the polaritonic fields, facilitating additional effects such as focusing of polaritons at designated positions through the use of aperiodic arrangements.

To excite strongly confined surface modes, we are interested in small scatterers compared to the light wavelength. This condition is hard to reconcile with the requirement of lossless particles, but some suitable choices can be found depending on the spectral range of interest. For example, we show that hBN disks are excellent resonant scatterers at mid-infrared frequencies, where they can sustain photon-polaritons with reasonably low material losses that are overshadowed by those associated with their coupling to SPs. Our results show that these particles can assist electron coupling to graphene plasmons under conditions for which direct coupling is kinematically forbidden. Mie modes in dielectric particles offer another possibility in a spectral range for which materials with a relatively high refractive index are available, such as silicon and germanium in the near-infrared. For visible frequencies, noble-metal nanoparticles can feature plasmonic resonances that only depend on morphology in the quasistatic limit (particle size much smaller than the light wavelength), so they can be made arbitrarily small down to a few nanometers (when nonlocal effects become relevant), although intrinsic losses in the material are substantial. We further envision arrays formed by optically trapped two-level atoms as an appealing approach to realize lossless resonant scatterers. Similar effects to the ones shown in this paper should be produced when using nondipolar, bulky scatterers, which can be of interest for experimental purposes. In that scenario, the assumed point-like approximation fails, so that full numerical simulations are required, which could be explored in future works.

The observation of Smith–Purcell radiation [76], [89] requires that electrons interact with many periods of a grating. State-of-the-art electron optics can produce energetic electrons collimated within a small angle (e.g., ∼10 μrad [90]), such that electrons propagate for a large distance before deviating significantly in the out-of-plane direction (e.g., ∼100 ’s μm in-plane distance for 1 nm out-of-plane deviation), thus interacting with many periods of the scatterer arrays. Under such conditions, strong collimation of the generated radiation (in-plane polaritons in the present work) should take place.

Unlike other methods of SP generation, the use of free electrons to excite SPs presents the advantage that no structuring of the surface is necessary, and although a spacer might be required to separate the scatterers from the SP-supporting interface, the latter still retains in-plane translational symmetry. Through the mediation of nanoparticles decorating the surface at an optimum distance, we show that SPs can be efficiently excited at places determined by the position of the particles and under conditions for which direct electron–polariton coupling is not possible when using low-energy electrons. In addition, resonant particles allow spectral selectivity of the generated polaritons, filtering specific frequencies from the broadband field provided by the moving electrons. For particle arrays, a directional emission of SPs is predicted, displaying interesting features associated with lattice resonances that deserve further exploration in future studies.

## 5 Methods

### 5.1 Optical response of the materials under consideration

The optical response of supported graphene is described through its surface conductivity in the Drude model [Eq. (10)], with *ω*_D_ = *E*_F_/*πℏ* depending on the doping Fermi energy *E*_F_. For silver, the permittivity can be well-approximated by a modified Drude model $\epsilon \left(\omega \right)=\epsilon_{\text{b}}-\omega_{\text{bulk}}^{2}/\omega \left(\omega +\text{i}\gamma \right)$ with parameters *ϵ*_b_ = 4, *ℏω*_bulk_ = 9.17 eV, and *ℏγ* = 21 meV extracted from optical measurements [82], [91]; in our calculations, we set *ϵ*_b_ = 1 for simplicity, as this parameter does not influence the results significantly for the plasmon frequencies under consideration. For thin hBN, polarization in the material is dominated by in-plane directions described by the corresponding permittivity component *ϵ*_hBN_(*ω*), and consequently, we ignore the out-of-plane permittivity and approximate [92] $\epsilon_{\text{hBN}}\left(\omega \right)=\epsilon_{\infty }-f\omega_{0}^{2}/\left[\omega \left(\omega +\text{i}\gamma \right)-\omega_{0}^{2}\right]$ with parameters *ϵ*_∞_ = 4.90, *f* = 2.00, *ℏω*_0_ = 168.6 meV, and *ℏγ* = 0.87 meV in the upper Reststrahlen band.

An effective surface conductivity *σ*(*ω*) = i*ωd*[1 − *ϵ*(*ω*)]/4*π* can be extracted from the material permittivity *ϵ*(*ω*) for a film of small thickness *d* compared with the polariton wavelength. We apply this approach to describe silver and hBN disks by setting *ϵ*(*ω*) to the respective permittivities of these materials (see above). For silver, this procedure leads to the Drude conductivity in Eq. (10) with $\omega_{\text{D}}=\hbar \omega_{\text{bulk}}^{2}d/4\pi e^{2}$ .

### 5.2 Effective polarizability of a thin disk near an embedded 2D layer

In the electrostatic limit, the in-plane polarizability of a thin disk of diameter *D* supported on a homogeneous substrate of permittivity *ϵ* can be expressed as [86]

(25a) $$\alpha_{\|}\left(\omega \right)=\bar{\epsilon }\;D^{3}\underset{j}{\sum }\frac{\zeta_{j}^{2}}{1/\eta \left(\omega \right)-1/\eta_{j}}$$

in terms of contributions arising from different polaritonic eigenmodes *j*, which enter through their associated dipolar matrix elements *ζ*_*j*_ and eigenvalues *η*_*j*_. Here, $\bar{\epsilon }=\left(\epsilon +1\right)/2$ is the average permittivity of the media on both sides of the disk, while $\eta \left(\omega \right)=i\sigma \left(\omega \right)/\bar{\epsilon }\omega D$ captures the response of the disk material through its surface conductivity *σ*(*ω*). We dismiss the out-of-plane polarizability (i.e., *α*_⊥_ = 0) under the assumption that the disk thickness *d* is small compared with *D*. The response is dominated by the lowest-order dipolar mode *j* = 1, for which one finds the parameters [86] *ζ*_1_(*x*) = *a*e^*bx*^ + *c* and *η*_1_(*x*) = *a*′e^*b*^^′^^*x*^ + *c*′, which depend on the thickness-to-diameter ratio *x* = *d*/*D* and is parametrized with material-independent constants *a* = −0.01267, *b* = −45.34, *c* = 0.8635, *a*′ = 0.03801, *b*′ = −8.569, and *c*′ = −0.1108. For hBN disks, we set the thickness to *d* = 1 nm (roughly 3 hexagonal atomic monolayers).

The polarizability in Eq. (25a) assumes a thick substrate of permittivity *ϵ*. However, under the configuration considered in Figures 2 and 4, a 2D material layer [surface conductivity *σ*(*ω*)] is placed at a distance *z*_0_ below the outer surface on which the disk is lying. This layer changes the polarizability to an effective value $\alpha_{\|}^{\text{eff}}\left(\omega \right)$ given by an expression analogous to Eq. (6b), but with $G_{\|}\left(\omega \right)$ reinterpreted as the field that is self-induced by the presence of the 2D layer on a unit parallel dipole placed at the position of the disk [i.e., the effect of the homogeneous substrate of permittivity *ϵ* is already included in Eq. (25a)]. Considering the p-polarization electrostatic reflection coefficient $r_{k_{\|}\text{p}}^{\prime }=k_{\|}/\left(k_{\|}-k_{\text{p}}^{\prime }\right)$ with $k_{\text{p}}^{\prime }=\text{i}\omega \epsilon /2\pi \sigma \left(\omega \right)$ for a 2D layer embedded in an infinite material of permittivity *ϵ* [78] [cf. this expression and Eq. (9) for a supported 2D layer], as well as *r*_0_ = (*ϵ* − 1)/(*ϵ* + 1) for the substrate alone, we find

(25b) $$\alpha_{\|}^{\text{eff}}\left(\omega \right)=\frac{1}{1/\alpha_{\|}\left(\omega \right)-{\tilde{G}}_{\|}\left(\omega \right)}$$

with *α*_‖_(*ω*) given by Eq. (25a) and

(25c) $${\tilde{G}}_{\|}\left(\omega \right)=\frac{2\epsilon }{{\left(\epsilon +1\right)}^{2}}\overset{\infty }{\underset{0}{\int }}k_{\|}^{2}\text{d}k_{\|}\;\frac{r_{k_{\|}\text{p}}^{\prime }{\text{e}}^{-2k_{\|}z_{0}}}{1+r_{0}r_{k_{\|}\text{p}}^{\prime }{\text{e}}^{-2k_{\|}z_{0}}}.$$

Compared to Eq. (7), this expression incorporates an overall factor 4*ϵ*/(*ϵ* + 1)^2^ arising from the forward and backward field transmission across the outer planar surface, as well as a denominator accounting for multiple scattering in the cavity defined by the surface and the 2D material. We use Eq. (25b) for the effective polarizability of the disk in Figures 2 and 4.

### 5.3 2D polariton emission probability by an induced dipole

We can obtain the SP emission probability from the energy absorbed by the 2D material in the configuration of Figure 1(a) under the assumption that the response is dominated by SPs. The energy absorbed by a material of permittivity *ϵ*_*m*_(*ω*) occupying a region *V* and exposed to an optical field **E**(**r**, *ω*) (in the frequency domain) is given by [77] $\int_{0}^{\infty }\text{d}\omega \;\hbar \omega \;\Gamma \left(\omega \right)$ , where

(26) $$\Gamma \left(\omega \right)=\frac{1}{4\pi^{2}\hbar }\underset{V}{\int }{\text{d}}^{3}r\;Im\left\{\epsilon_{m}\left(\omega \right)\right\}\;|E\left(r,\omega \right){|}^{2}.$$

For the 2D material, we consider an arbitrarily small thickness *d*, which yields a high permittivity *ϵ*_*m*_(*ω*) = 1 + 4*π*i*σ*(*ω*)/*ωd* expressed in terms of the surface conductivity *σ*(*ω*). The normal electric field inside the material is therefore negligible because of the continuity of the normal displacement, while the parallel field created by the dipole at the surface plane is continuous and given by Eqs. (4a) and (4b) with *z* = −*z*_0_. In the electrostatic limit, this field reduces to

(27) $$\begin{array}{ll} E_{\text{dip}\|}\left(R,-z_{0},\omega \right) & =\frac{-i}{2\pi }\int {\text{d}}^{2}k_{\|}\;{\text{e}}^{\text{i}k_{\|}\cdot R-k_{\|}z_{0}}\\ & \;\times \;\left(r_{k_{\|}\text{p}}-1\right)\;\left[p\left(\omega \right)\cdot \left(i{\hat{k}}_{\|}+\hat{z}\right)\right]\;k_{\|}. \end{array}$$

Inserting this result into Eq. (26), carrying out the **R** integral analytically, and reducing the *z* integral to a factor *d*, we find

$$\begin{array}{ll} \Gamma \left(\omega \right) & =\frac{1}{\hbar \omega }Re\left\{\sigma \left(\omega \right)\right\}\\ & \;\times \overset{\infty }{\underset{0}{\int }}k_{\|}^{3}\text{d}k_{\|}\;{\text{e}}^{-2k_{\|}z_{0}}|r_{k_{\|}\text{p}}-1{|}^{2}\left[|p_{\|}{|}^{2}+2|p_{z}{|}^{2}\right], \end{array}$$

which, upon consideration of Eq. (9) and the dispersion relation $k_{\text{p}}=i\omega \bar{\epsilon }/2\pi \sigma \left(\omega \right)$ for the supported film in Figure 1(a), finally transforms into Eq. (14) with $Im\left\{G_{\|}\left(\omega \right)\right\}$ defined by Eq. (7). To obtain this result, we have used the expressions $Re\left\{\sigma \left(\omega \right)\right\}=\left(\omega \bar{\epsilon }/2\pi \right)Im\left\{-1/k_{\text{p}}\right\}$ and $k_{\|}|r_{k_{\|}\text{p}}-1{|}^{2}=R_{\text{p}}Im\left\{r_{k_{\|}\text{p}}\right\}/Im\left\{-1/k_{\text{p}}\right\}$ (with $R_{\text{p}}=1/\bar{\epsilon }$ ) derived from the relations between *k*_p_, *σ*(*ω*), and $r_{k_{\|}\text{p}}$ .

For the SP emission probability in the configuration of Figure 2(a), the in-plane field acting on the 2D layer takes a similar form as Eq. (27), but now setting *p*_*z*_ = 0 for the disk, replacing $r_{k_{\|}\text{p}}$ by the reflection coefficient $r_{k_{\|}\text{p}}^{\prime }$ of the fully *ϵ*-embedded 2D film (see Section 5.2), and inserting an additional factor $t_{0}/\left(1+r_{0}r_{k_{\|}\text{p}}^{\prime }{\text{e}}^{-2k_{\|}z_{0}}\right)$ in the integrand to account for the transmission of the dipole field across the topmost surface [transmission coefficient $t_{0}=2\sqrt{\epsilon }/\left(\epsilon +1\right)$ ] as well as multiple Fabry–Perot scattering within the surface–2D-film cavity. Then, by following the same steps as above but using the so-modified field, we trivially obtain Eq. (18) with the Green tensor defined in Eq. (19).

### 5.4 Dipole–dipole interaction in linear arrays

In a linear array, the dipole self-interaction needs to be supplemented by the contribution of the field induced by other dipoles. We thus redefine $G\left(\omega \right)=G^{\text{dir}}\left(\omega \right)+G^{\text{ref}}\left(\omega \right)$ as the sum of a direct term contributed by all other dipoles in the array plus a surface-reflected term produced by all dipoles. Starting from Eqs. (4a) and (4b) and adopting the electrostatic limit, the modified Green tensor is found to have vanishing off-diagonal components. The direct contribution can easily be evaluated by numerically summing the analytical expression for the free-space dipole–dipole interaction, yielding $G_{xx}^{\text{dir}}\left(\omega \right)=2S^{\text{dir}}$ and $G_{yy}^{\text{dir}}\left(\omega \right)=G_{zz}^{\text{dir}}\left(\omega \right)=-S^{\text{dir}}$ with

$$S^{\text{dir}}=\frac{2}{a^{3}}\overset{\infty }{\underset{j=1}{\sum }}\frac{\cos\left(j\omega a/v\right)}{j^{3}}.$$

For the surface-reflected components, we follow a procedure similar to the derivation of Eq. (22) and write

(28) $$\begin{array}{ll} & \;\left[G_{xx}^{\text{surf}}\left(\omega \right),G_{yy}^{\text{surf}}\left(\omega \right),G_{zz}^{\text{surf}}\left(\omega \right)\right]\\ & =\frac{1}{a}\overset{\infty }{\underset{n=-\infty }{\sum }}\overset{\infty }{\underset{-\infty }{\int }}\frac{\text{d}k_{y}}{k_{n\|}}\;\left[k_{nx}^{2},k_{y}^{2},k_{n\|}^{2}\right]\;{\text{e}}^{-2k_{n\|}z_{0}}r_{k_{n\|}\text{p}}, \end{array}$$

with *k*_*nx*_ = *ω*/*v* + 2*πn*/*a* and $k_{n\|}=\sqrt{k_{nx}^{2}+k_{y}^{2}}$ . Now, we consider low-loss SPs and a surface reflection coefficient satisfying Eq. (11), which allows us to evaluate the *k*_*y*_ integral and write

(29) $$\begin{array}{ll} & \;Im\left\{\left[G_{xx}^{\text{surf}}\left(\omega \right),G_{yy}^{\text{surf}}\left(\omega \right),G_{zz}^{\text{surf}}\left(\omega \right)\right]\right\}\\ & \approx \frac{2\pi R_{\text{p}}}{a}\overset{\infty }{\underset{n=-\infty }{\sum }}\frac{k_{\text{p}}}{\sqrt{k_{\text{p}}^{2}-k_{nx}^{2}}}\;\left[k_{nx}^{2},k_{\text{p}}^{2}-k_{nx}^{2},k_{\text{p}}^{2}\right]\;{\text{e}}^{-2k_{\text{p}}z_{0}}, \end{array}$$

for the surface-reflected component of the Green tensor in the array, where $R_{\text{p}}=1/\bar{\epsilon }$ and *k*_p_ are approximated as real numbers and the *n* sum is restricted by |*k*_*nx*_| < *k*_p_ (see Supplementary Materials, Figure S5).

## Supplementary Material

Supplementary Material Details
