# Supplementary material for: Free-electron coupling to surface polaritons mediated by small scatterers
Source: Nanophotonics. 2024 Oct 4;13(25):4667–81. doi: 10.1515/nanoph-2024-0326 (PMC11614564; doi:10.1515/nanoph-2024-0326)
Supplement: Supplementary file 1 — Supplementary Material Details [file j_nanoph-2024-0326_suppl_001.pdf]

Free-electron coupling to surface polaritons mediated by small scatterers

– SUPPLEMENTARY INFORMATION –

L. Prelat,[1] E. J. C. Dias,[1] and F. Javier García de Abajo[1,2,*]

[1]*ICFO-Institut de Ciencies Fotoniques, The Barcelona Institute of Science and Technology, 08860 Castelldefels (Barcelona), Spain*

[2]*ICREA-Institució Catalana de Recerca i Estudis Avançats, Passeig Lluís Companys 23, 08010 Barcelona, Spain*

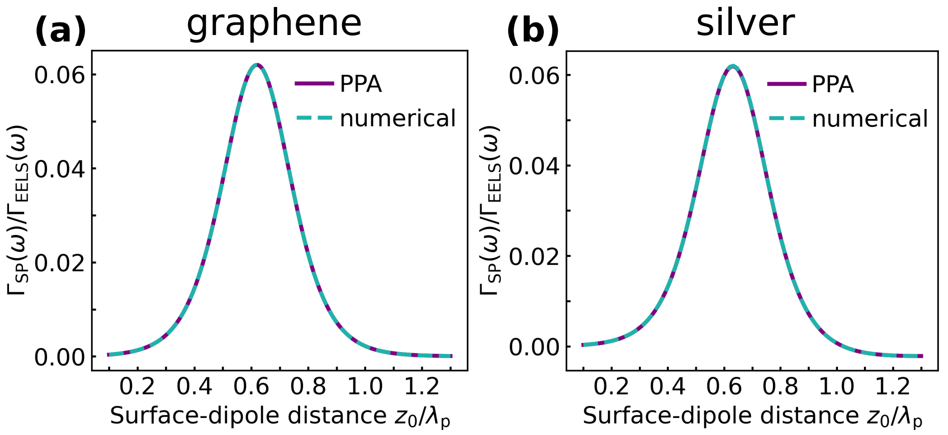

FIG. S1. **Electron coupling to surface polaritons mediated by a small scatterer: numerical vs analytical approaches**. Probability of polariton emission $\Gamma_{SP}(\omega)$ normalized to the EELS probability $\Gamma_{EELS}(\omega)$ as a function of scatterer–surface distance z_0 for **(a)** graphene (emission energy $\hbar\omega = 120\,$meV, Fermi energy $E_F = 1$ eV) and **(b)** silver ($\hbar\omega = 1.75\,$eV). The probability is calculated either numerically (dashed-light-blue curves) or analytically through the polariton-pole approximation (PPA, purple curves). In (b), we consider a 1 nm-thick film. We set $v = 0.04c$ and $k_D b = 0.53$ in both panels.

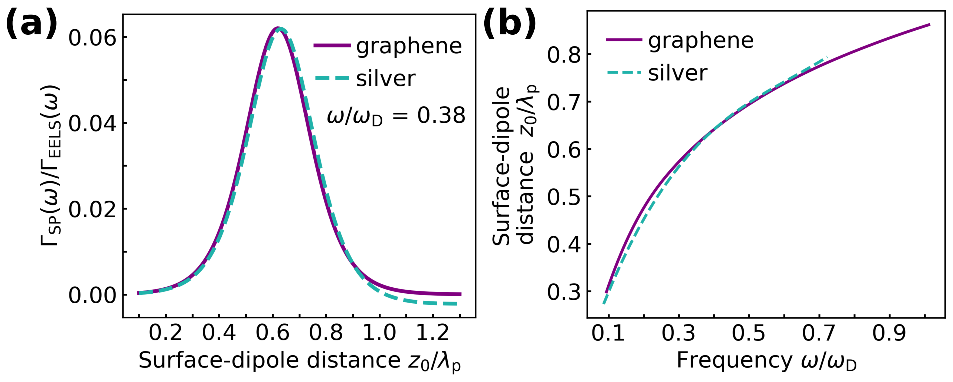

FIG. S2. **Electron coupling to surface polaritons mediated by a small scatterer: graphene vs silver**. **(a)** Probability of surface-plasmon emission $\Gamma_{SP}(\omega)$ normalized to the EELS probability $\Gamma_{EELS}(\omega)$ for a graphene monolayer (Fermi energy $E_F = 1$ eV) and a silver film (thickness $d = 1$ nm) as a function of the scattered–surface distance z_0 normalized to the corresponding plasmon wavelength λ_p for a fixed emission frequency $\omega = 0.38\,\omega_D$. **(b)** Optimum surface–dipole distance that maximizes $\Gamma_{SP}(\omega)/\Gamma_{EELS}(\omega)$ as a function of frequency for the same materials as in (a). We set $v = 0.04c$ and $k_D b = 0.53$ in both panels.

* javier.garciadeabajo@nanophotonics.es

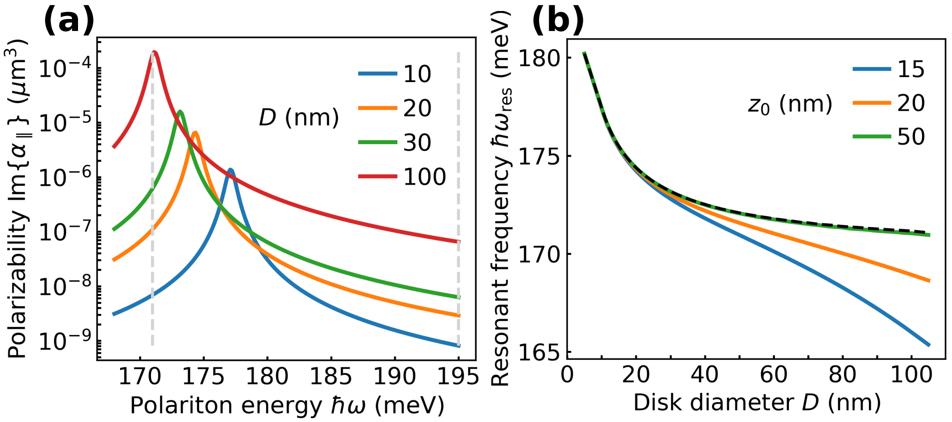

FIG. S3. **Size-dependence of hBN disk polarizabilities.** **(a)** Imaginary part of the in-plane polarizability of hBN disks of 1 nm thickness and different diameters D (see labels) supported on a homogeneous substrate of permittivity $\epsilon = 2$, as calculated from Eq. (25a) in the main text. **(b)** Peak energy extracted from (a) as a function of disk size (dashed-black curve), compared with the peak energy obtained from the effective disk polarizability under the configuration of Fig. 2a [Eq. (25b)] for different separations z_0 (see labels). We set the graphene Fermi energy to $E_F = 1$ eV and the hBN disk thickness to $d = 1$ nm.

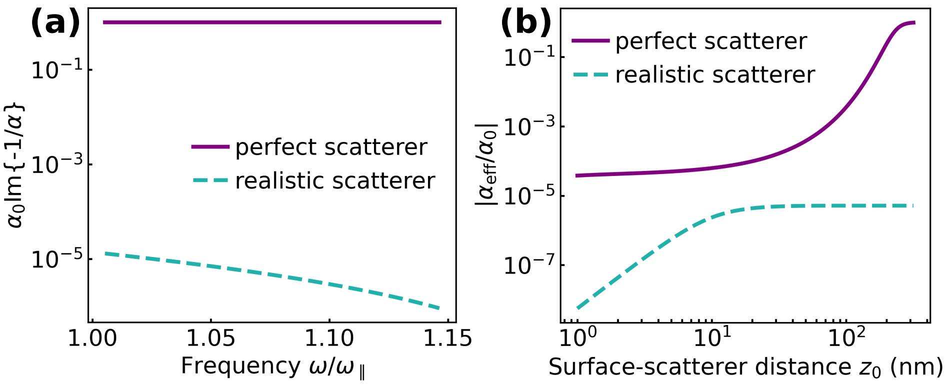

FIG. S4. **Effective polarizability of an hBN disk and a perfect (lossless and resonant) dipole**. **(a)** Frequency dependence of the imaginary part of the inverse polarizabilities Im$\{-1/\alpha\}$ for a perfectly scattering dipole [$\alpha = i\alpha_0$ with $\alpha_0 = 3\epsilon/2k_1^3$ and $\epsilon = 2$] and an hBN disk [Eq. (25a) in the main text], normalized to $1/\alpha$. The frequency is normalized to the in-plane resonant frequency of hBN, $\omega_\parallel = 171.1$ meV. **(b)** Absolute value of the in-plane component of the effective polarizability α_{eff} given by Eq. (25b) in the main text and normalized to α_0 for the same two scatterers as in (a), but placed at a distance z_0 from a graphene layer (Fermi energy $E_F = 1$ eV). We present results as a function of z_0 for fixed energy $\hbar\omega = 180$ meV. The hBN disk thickness and diameter are $d = 1$ nm and $D = 20$ nm in all cases.

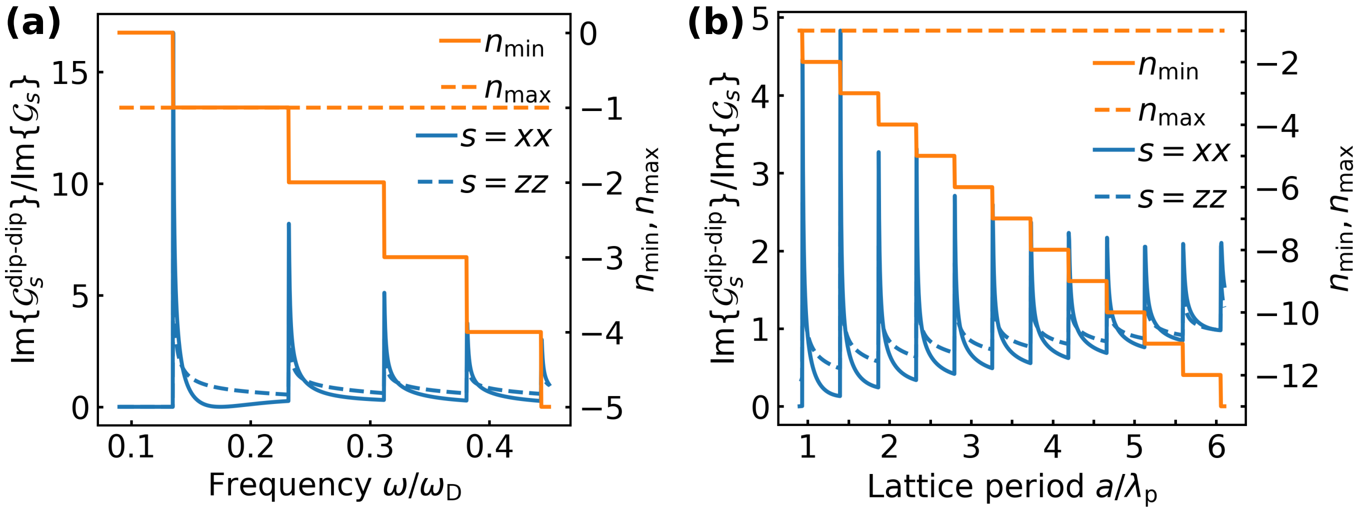

FIG. S5. **Lattice resonances in the dipole–dipole interaction of linear periodic arrays**. **(a)** Frequency dependence of the ratio $\mathrm{Im}\{\mathcal{G}_s^{\rm surf}\}/\mathrm{Im}\{\mathcal{G}_s^{\rm particle}\}$ between the Green tensor components of a particle array [only the surface contribution $\mathrm{Im}\{\mathcal{G}_s^{\rm surf}\}$, calculated from Eq. (29) in the main text] and a single particle [$\mathrm{Im}\{\mathcal{G}_s^{\rm particle}\}$, taken from Eq. (12)] for $s = xx$ (equivalent to $\parallel$ in the single particle) and $s = zz$ ($\perp$ in the single particle). The frequency is normalized to $\omega_{\rm D}$. In the array, the ratio of the period to the polariton wavelength is set to $a/\lambda_{\rm p} = 2$. **(b)** Same ratio of Green-tensor components as in panel (a), plotted as a function of $a/\lambda_{\rm p}$ for a fixed frequency $\omega = 0.4\,\omega_{\rm D}$. We use the parameters $z_0 = 0.6\,\lambda_{\rm p}$, $k_{\rm D}b = 0.1$, and $v = 2.2v_0$ in both panels. The right axes indicate the upper and lower limits of the sum in Eq. (29): $n_{\rm max}$ (dashed-orange curve) and $n_{\rm min}$ (solid-orange curve), respectively.
